# Malyngamide F Possesses Anti-Inflammatory and Antinociceptive Activity in Rat Models of Inflammation

**DOI:** 10.1155/2021/4919391

**Published:** 2021-06-18

**Authors:** Zhuocheng Li, Lei Zhang, Zhichao Zhao

**Affiliations:** ^1^Department of Anesthesiology, Heilongjiang Provincial Hospital, No. 82 Zhongshan Road, Xiangfang District, Harbin City, Heilongjiang Province 150036, China; ^2^Department of Anesthesiology, Harbin Chest Hospital, No. 417 Xianfeng Road, Daowai District, Harbin City, Heilongjiang Province 150026, China; ^3^Operation Room, Heilongjiang Provincial Hospital, No. 82 Zhongshan Road, Xiangfang District, Harbin City, Heilongjiang Province 150036, China

## Abstract

**Objective:**

Inflammation and pain are involved in the pathophysiology of various clinical conditions. This investigation aims to probe the analgesic and anti-inflammatory activity of Maltoamide F.

**Methods:**

The possible toxicity of Maltoamide F was evaluated by an acute toxicity test. To assess the anti-inflammatory and antinociceptive effects of Maltoamide F on rats, the models of carrageenan-caused paw edema, xylene-induced ear edema, arachidonic-acid- (AA-) induced ear edema, formalin-caused plantar edema, and cotton-pellet-induced granuloma were established. Levels of TNF-*α*, PGE-2, and IL-6 were detected by enzyme-linked immunosorbent assay (ELISA).

**Results:**

Maltoamide F was safe at oral doses of 1–10 mg/kg for rats. Maltoamide F (1 mg/kg, 5 mg/kg, and 10 mg/kg) notably reduced carrageenan-induced edema percentage of paws in rats and decreased levels of PGE-2, IL-6, and TNF-*α* in homogenates of foot tissues. Maltoamide F (1 mg/kg, 5 mg/kg, and 10 mg/kg) reduced levels of PGE-2, IL-6, and TNF-*α* in foot tissues of formalin-induced rats. Maltoamide F (1 mg/kg, 5 mg/kg, and 10 mg/kg) repressed AA-induced increase of ear thickness in rats and reduced levels of PGE-2, IL-6, and TNF-*α* in homogenates of ear tissues. Maltoamide F (1 mg/kg, 5 mg/kg, and 10 mg/kg) reduced xylene-induced weight of ear edema in rats and reduced levels of PGE-2, IL-6, and TNF-*α* in homogenates of ear tissues. Maltoamide F (1 mg/kg, 5 mg/kg, and 10 mg/kg) reduced levels of PGE-2, IL-6, and TNF-*α* in homogenates of cotton ball granuloma of cotton-pellet-induced rats.

**Conclusions:**

Maltoamide F possessed anti-inflammatory and analgesic activity in inflammatory models of rats.

## 1. Introduction

Inflammation is a natural response of the body to detrimental stimuli, which occurs through incremental movement of leucocytes from blood to damaged tissues [1]. It is developed by the release of cytokines, bradykinin, and histamine [2, 3]. Swelling, redness, heat, and pain are the major features of inflammation [4, 5]. The controlled inflammation can remove the irritants, eliminate invasive organisms, and promote the repair of damaged tissues [6, 7]. However, uncontrolled inflammation leads to organ dysfunction and tissue damage, thereby causing inflammatory diseases [8–10]. At present, common treatments used to treat inflammatory diseases are nonsteroidal and steroidal drugs, but side effects of these drugs limit their clinical applications [11, 12]. Thence, there is an urgent need to search new anti-inflammatory agents with less toxicity and good efficacy.

Malyngamides are a type of metabolite from *L. majuscula* [13, 14]. Structurally, malyngamides are composed of an assumed amino-acid-derived head and a methoxylated fatty acid tail [15]. Since 1978, over 30 different malyngamides have been isolated, which have a wide range of biological properties such as antimicrobial, antimycobacterial, and anti-inflammatory properties [16–21]. Importantly, several members of malyngamides are uncovered to possess anti-inflammatory effects [22, 23]. For instance, Villa et al. have unveiled that Malyngamide F acetate displays anti-inflammatory activity in the NO assay with no cytotoxicity at the concentrations tested [22]. Malloy et al. have revealed that Malyngamide 2 shows anti-inflammatory activity in LPS-induced macrophage cells [23]. However, the anti-inflammatory role of Malyngamide F has not been verified in animal models.

Hereon, we intended to determine the toxicity of Malyngamide F in rats and further assessed the anti-inflammatory activity and analgesic activity of Malyngamide F in rat models of inflammation.

## 2. Materials and Methods

### 2.1. Animals

Male Sprague Dawley (SD) rats (weighing 210–260 g) were bought from Esebio (Shanghai, China). All rats were housed in a controlled environment with a temperature of 21 ± 2°C and a cycle of 12 h light/dark. Before the experiment, rats had free access to water and food. This investigation was approved by the Animal Care and Use Committee of our hospital, and all experiments were executed on the basis of the National Institutes of Health Guide for the Care and Use of Laboratory Animals. Each experimental group is composed of 6 rats, and each rat was used only once in our experiments.

### 2.2. Drugs and Reagents

Indomethacin F was bought from Chengdu Pharmaceutical Factory (Chengdu, China), and carrageenan, formalin, xylene, and arachidonic acid (AA) were purchased from Sigma Chemical Co. (St. Louis, MO, USA).

### 2.3. Collection of Materials

The marine cyanobacterium *Lyngbya majuscula* was collected from Kahala Beach, Oahu island, Hawaii, and stored at −20°C until workup.

### 2.4. Extraction and Isolation

For obtaining 663 mg of cytotoxic crude organic extract, cyanobacterium *L. majuscula* (19.3 g) was isolated by CH_2_Cl_2_ : MeOH (2 : 1, v/v). To obtain nine fractions, this extract was fractionated via vacuum liquid chromatography (VLC). Fractions (5, 6, and 7) displayed cytotoxic impacts and then were further explored. To obtain two definite fractions, fraction 5 was resolved with RP-HPLC. Then, a second chromatography of the first fraction generated 24.6 mg of 8-*O*-acetyl-8-*epi*-malyngamide C (1). Further separation of VLC fraction 6 led to additional compound 1 (9.5 mg) and an indivisible mixture of malyngamide H (6) and K (8). Next, reversed-phase HPLC of VLC fraction 7 generated 1.9 mg 6-*O*-acetyl malyngamide F (5), 0.8 mg compound 3, and 1.1 mg inseparable mixture of malyngamide H (6) and K (8). ^1^H NMR profiling of all VLC fractions indicated that fraction 8 also included a malyngamide-type metabolite. Thus, fraction 8 was first subjected to C_18_ HPLC, and those materials were purified through RP-HPLC to obtain malyngamide J (7).

### 2.5. Drug Treatment

The rats in three groups (*n* = 6) were given Maltoamide F (1, 5, and 10 mg/kg) by intubation. Rats in the positive control group (*n* = 6) were given indomethacin (5 mg/kg), and rats in the negative control group (*n* = 6) were treated with 1% EtOH. All rats were given drugs at a dose of 10 mL/kg according to their body weight.

### 2.6. Acute Toxicity Test

The toxicity of Malyngamide F was evaluated by the acute toxicity test. Rats were treated via oral administration of extracts in different doses, and the dose was adjusted according to rat response [24]. The maximum dose given to each rat was 50 mg/kg, and rats in the control group were treated with distilled water. All rats were observed within 7 days.

### 2.7. Anti-Inflammatory Experiments

In the paw edema test [25], 0.1 mL carrageenan (1%) in normal saline was injected into the right hind paws of rats to induce inflammation. At 0, 0.5, 1, 2, 3, 4, and 6 h after carrageenan injection, the paw volume was gauged with a paw edema detector (Shandong Academy of Medical Science Device Station, Shandong, China). Rats were orally administered either indomethacin or Malyngamide F before 0.5 h of carrageenan injection. The calculation of edema percentage was as follows: edema percentage (%) = (*V*_*t*_ − *V*_0_)/*V*_0_ × 100, where *V*_0_ is the volume before carrageenan injection and *V*_*t*_ is the volume at *t* h after carrageenan injection.

In the plantar edema test [26], 20 *μ*L formalin (2%) was injected into the right hind paws of rats to induce inflammation [27]. Before formalin injection for 1 h, Maltamide F or indomethacin was orally administered for 7 consecutive days. Mean increase of paw volume was gauged on the first and seventh day, and the percent inhibition was calculated with the following formula [28]: inhibition (%) = (*V*_*c*_ − *V*_*t*_)/*V*_*c*_ × 100, where *V*_*c*_ is the mean increase in edema in the control group and *V*_*t*_ is mean increase in edema in treated groups.

In the ear edema test [29], AA was dissolved in acetone at a concentration of 100 mg/mL, and then, 20 *μ*L AA (2 mg/ear) was used to treat each rat on both surfaces of an ear. At 30 min before AA induction, Maltamide F and indomethacin were dissolved in ethanol and were suspended with 1% polyoxyethylene sorbitan monooleate (Tween 80, Tokyo Kasei Chemical Industry, Japan). After AA induction for 1 h, ear edema was gauged by using a dial thickness gauge (Ozaki Factory, Japan).

In the xylene-induced ear edema assay [30], rats were treated with Maltamide F and indomethacin for 1 h. Subsequently, the surface of the right ear was treated by 30 *μ*L xylene for 30 min, and then, both ears were removed. Punch biopsy specimens of a 6 mm^2^ ear were collected and weighed. The weight of ear edema was calculated via subtracting the weight of the left ear from that of the right.

In the cotton pellet granuloma test [31], rats were anaesthetized via ethyl ether, and then, the back skin was scraped and disinfected with 70% ethanol. An incision was generated in the lumbar region. Next, a subcutaneous tunnel was generated and a cotton pellet was placed in the scapular region. After surgery, each rat was intramuscularly injected with benzathine penicillin (60,000 IU). The rats in each group were treated with maltamide F or indomethacin for seven days. On the 8^th^ day, the rats were sacrificed and the cotton pellets were removed through surgery. After removing extraneous tissues, moist pellets were weighed (wet weight). Then, the pellets were dried at 90°C for 3 h, and the dried pellets were weighed (dry weight).

### 2.8. Enzyme-Linked Immunosorbent Assay (ELISA)

Ear tissues, foot tissues, and cotton ball granuloma were collected and then homogenized in PBS containing 1% protease inhibitor. Afterwards, the homogenates were centrifuged at 10,000 g and placed at 4°C for 30 min. Finally, the content of tumor necrosis factor-a (TNF-*α*), interleukin-6 (IL-6), and PGE-2 (a pain-related factor) was measured by using Quantikine ELISA Kits (R&D Systems, Abingdon, UK). The optical density was examined at 450 nm using a microplate reader (Molecular Devices, Sunnyvale, CA).

### 2.9. Statistical Analysis

SPSS 21.0 software (IBM Corp, Armonk, NY, USA) was employed to analyze data. All results were exhibited by means ± standard deviation. Differences among multigroups were compared with one-way analysis of variance (ANOVA), followed by Tukey's multiple comparisons. *P* < 0.05 indicated a statistically significant difference.

## 3. Results

### 3.1. Maltoamide F Is Safe at Oral Doses of 1–10 mg/kg for Rats

**Figure 1:**
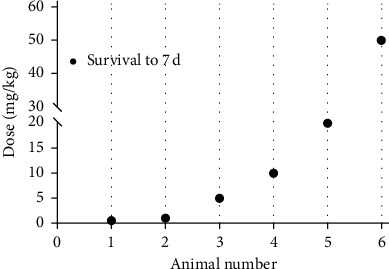
Maltoamide F is safe for rats at oral doses of 1–10 mg/kg, the acute toxicity test of Maltoamide F in rats.

To determine the oral dose of Maltoamide F, an acute toxicity test was implemented in rats. As displayed in Figure 1, the maximum oral dose of Maltoamide F was 50 mg/kg, and no death was observed during the evaluation period of 7 d. Therefore, Maltoamide F is safe at oral doses of 1–10 mg/kg for rats.

### 3.2. Maltoamide F Alleviated Carrageenan-Induced Paw Edema and Inflammation in Rats

**Figure 2:**
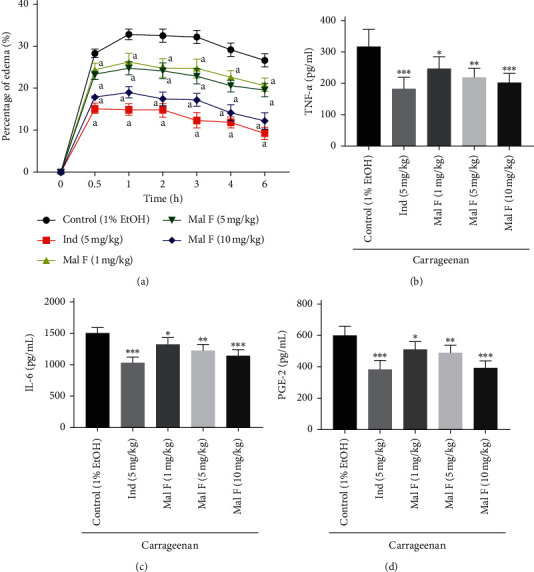
Maltoamide F alleviated carrageenan-induced paw edema and inflammation in rats. (a) The effect of Maltoamide F on percentage of paw edema in carrageenan-induced rats. a: *P* < 0.05 vs. control (1% EtOH). (b) The level of TNF-*α* in homogenates of foot tissues was measured by ELISA. ^*∗*^*P* < 0.05, ^*∗∗*^*P* < 0.01, and ^*∗∗∗*^*P* < 0.001 vs. control (1% EtOH). (c) The level of IL-6 in homogenates of foot tissues was measured by ELISA. ^*∗*^*P* < 0.05, ^*∗∗*^*P* < 0.01, and ^*∗∗∗*^*P* < 0.001 vs. control (1% EtOH). (d) The level of PGE-2 in homogenates of foot tissues was measured by ELISA. ^*∗*^*P* < 0.05, ^*∗∗*^*P* < 0.01, and ^*∗∗∗*^*P* < 0.001 vs. control (1% EtOH).

For exploring whether Maltoamide F made anti-inflammatory impacts on rats, we performed the following anti-inflammatory assays. We found that Maltoamide F distinctly reduced edema percentage of rat paws as opposed to the control group, which persisted for 6 h (*P* < 0.05, Figure 2(a)). Meantime, we discovered that 1 mg/kg Maltoamide F (*P* < 0.05), 5 mg/kg Maltoamide F (*P* < 0.01), and 10 mg/kg Maltoamide F (*P* < 0.001) markedly decreased the levels of PGE-2, IL-6, and TNF-*α* in comparison to the control group (Figures 2(b)–2(d)).

### 3.3. Maltoamide F Mitigated Formalin-Induced Inflammation in Rats

**Figure 3:**
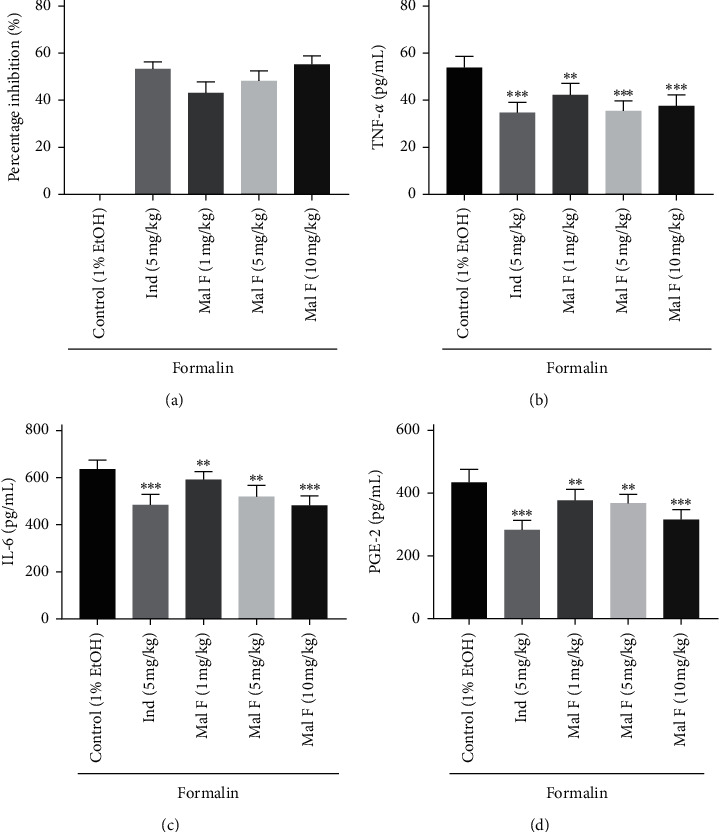
Maltoamide F mitigated formalin-induced inflammation in rats. (a) The effect of Maltoamide F on percentage inhibition in formalin-induced rats. (b) The level of TNF-*α* in homogenates of foot tissues was measured by ELISA. ^*∗∗*^*P* < 0.01 and ^*∗∗∗*^*P* < 0.001 vs. control (1% EtOH). (c) The level of IL-6 in homogenates of foot tissues was measured by ELISA. ^*∗∗*^*P* < 0.01 and ^*∗∗∗*^*P* < 0.001 vs. control (1% EtOH). (d) The level of PGE-2 in homogenates of foot tissues was measured by ELISA. ^*∗∗*^*P* < 0.01 and ^*∗∗∗*^*P* < 0.001 vs. control (1% EtOH).

Besides, we discovered that the percentage inhibition of 1 mg/kg and 5 mg/kg Maltoamide F was lower than that of 5 mg/kg indomethacin (Figure 3(a)). The 10 mg/kg Maltoamide F presented a comparable effect when compared to 5 mg/kg indomethacin (Figure 3(a)). Injection of 1 mg/kg (*P* < 0.01), 5 mg/kg (*P* < 0.01), and 10 mg/kg (*P* < 0.001) Maltoamide F distinctly reduced the levels of TNF-*α*, PGE-2, and IL-6 in homogenates of foot tissues (Figures 3(b)–3(d)).

### 3.4. Maltoamide F Lightened AA-Induced Ear Edema and Inflammation in Rats

**Figure 4:**
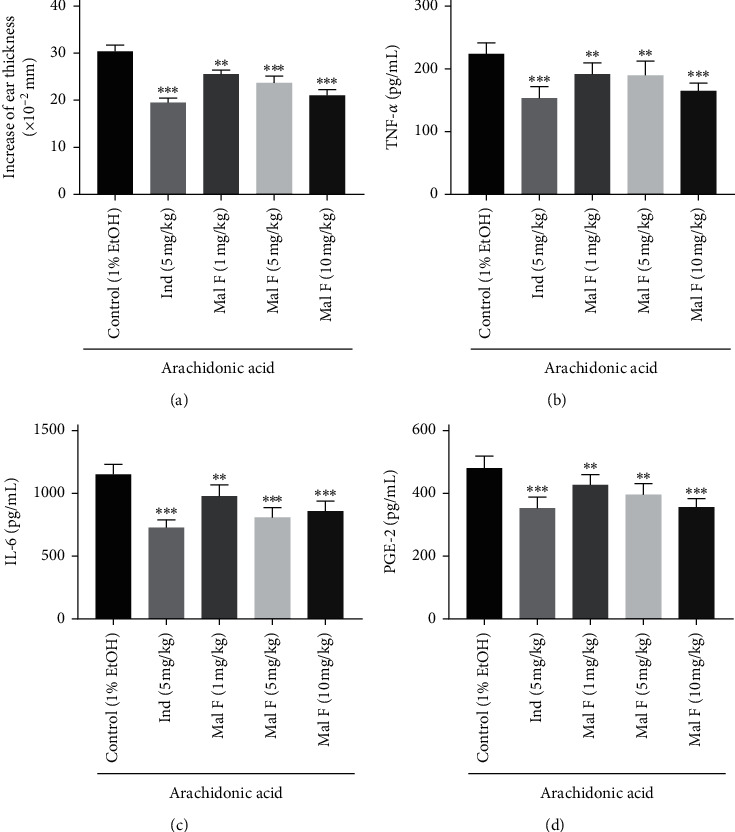
Maltoamide F lightened arachidonic-acid- (AA-) induced ear edema and inflammation in rats. (a) The effect of Maltoamide F on increase of ear thickness in AA-induced rats. ^*∗∗*^*P* < 0.01 and ^*∗∗∗*^*P* < 0.001 vs. control (1% EtOH). (b) The level of TNF-*α* in homogenates of ear tissues was measured by ELISA. ^*∗∗*^*P* < 0.01 and ^*∗∗∗*^*P* < 0.001 vs. control (1% EtOH). (c) The level of IL-6 in homogenates of foot tissues was measured by ELISA. ^*∗∗*^*P* < 0.01 and ^*∗∗∗*^*P* < 0.001 vs. control (1% EtOH). (d) The level of PGE-2 in homogenates of foot tissues was measured by ELISA. ^*∗∗*^*P* < 0.01 and ^*∗∗∗*^*P* < 0.001 vs. control (1% EtOH).

In contrast to the control group, 1 mg/kg (*P* < 0.01), 5 mg/kg (*P* < 0.001), and 10 mg/kg (*P* < 0.001) Maltoamide F repressed increase of ear thickness in rats (Figure 4(a)). Additionally, 1 mg/kg (*P* < 0.01), 5 mg/kg (*P* < 0.01), and 10 mg/kg (*P* < 0.001) Maltoamide F distinctly reduced levels of TNF-*α* and PGE-2, and the IL-6 level was also diminished by 1 mg/kg (*P* < 0.01), 5 mg/kg (*P* < 0.001), and 10 mg/kg (*P* < 0.001) Maltoamide F in homogenates of ear tissues of AA-induced rats (Figures 4(b)–4(d)).

### 3.5. Maltoamide F Alleviated Ear Edema and Inflammation Induced by Xylene in Rats

**Figure 5:**
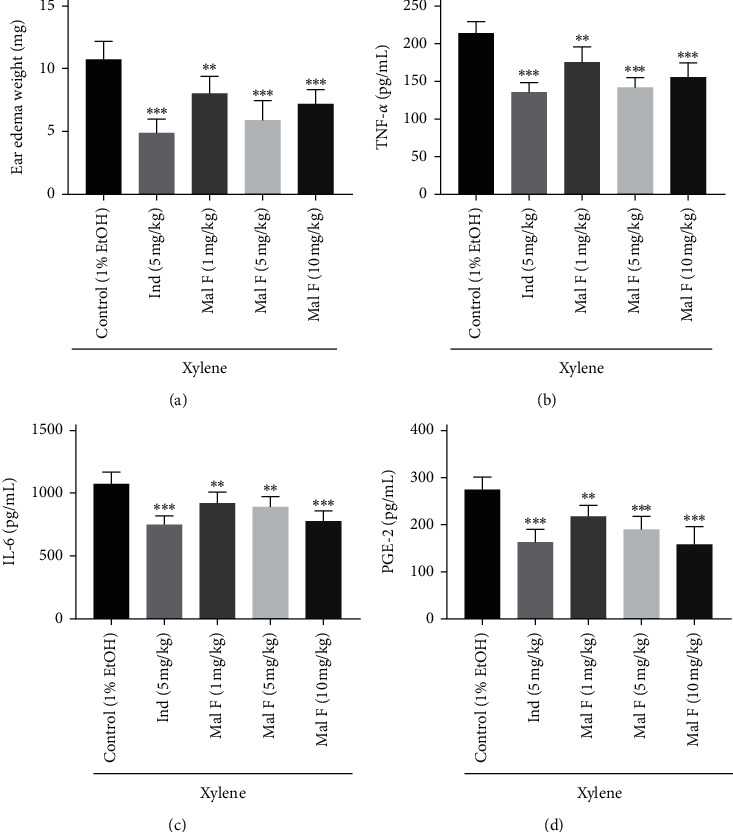
Maltoamide F alleviated ear edema and inflammation induced by xylene in rats. (a) Effects of Maltoamide F on xylene-induced ear edema in rats. ^*∗∗*^*P* < 0.01 and ^*∗∗∗*^*P* < 0.001 vs. control (1% EtOH). (b) The level of TNF-*α* in homogenates of ear tissues was measured by ELISA. ^*∗∗*^*P* < 0.01 and ^*∗∗∗*^*P* < 0.001 vs. control (1% EtOH). (c) The level of IL-6 in homogenates of foot tissues was measured by ELISA. ^*∗∗*^*P* < 0.01 and ^*∗∗∗*^*P* < 0.001 vs. control (1% EtOH). (d) The level of PGE-2 in homogenates of foot tissues was measured by ELISA. ^*∗∗*^*P* < 0.01 and ^*∗∗∗*^*P* < 0.001 vs. control (1% EtOH).

Compared with the control group, injection of 1 mg/kg, 5 mg/kg, and 10 mg/kg Maltoamide F markedly reduced ear edema weight in xylene-induced rats (Figure 5(a)). Moreover, 1 mg/kg (*P* < 0.01), 5 mg/kg (*P* < 0.001), and 10 mg/kg (*P* < 0.001) Maltoamide F distinctly reduced levels of TNF-*α* and PGE-2, and the IL-6 level was also diminished by 1 mg/kg (*P* < 0.01), 5 mg/kg (*P* < 0.01), and 10 mg/kg (*P* < 0.001) Maltoamide F in homogenates of ear tissues of xylene-induced rats (Figures 5(b)–5(d)).

### 3.6. Maltoamide F Mitigated Cotton-Pellet-Induced Granuloma and Inflammation in Rats

**Figure 6:**
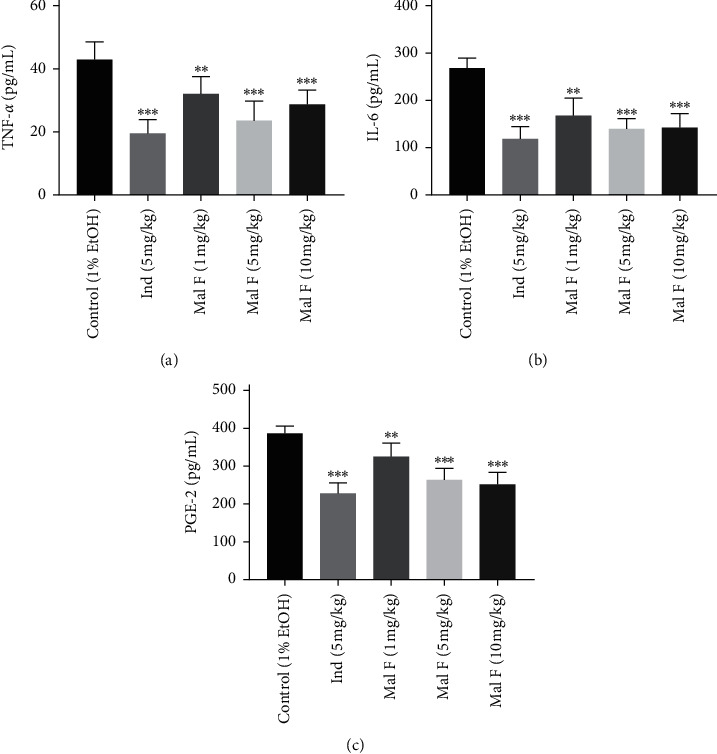
Maltoamide F alleviated ear edema and inflammation induced by the cotton pellet in rats. (a) The level of TNF-*α* in homogenates of cotton ball granuloma tissues was measured by ELISA. ^*∗∗*^*P* < 0.01 and ^*∗∗∗*^*P* < 0.001 vs. control (1% EtOH). (b) The level of IL-6 in homogenates of foot tissues was measured by ELISA. ^*∗∗*^*P* < 0.01 and ^*∗∗∗*^*P* < 0.001 vs. control (1% EtOH). (c) The level of PGE-2 in homogenates of foot tissues was measured by ELISA. ^*∗∗*^*P* < 0.01 and ^*∗∗∗*^*P* < 0.001 vs. control (1% EtOH).

Finally, it was demonstrated that the granuloma weight was significantly diminished by Maltoamide F (1 mg/kg, 5 mg/kg, and 10 mg/kg) in cotton-pellet-induced rats (all *P* < 0.05, Table 1). The levels of PGE-2, IL-6, and TNF-*α* were decreased by 1 mg/kg (*P* < 0.01), 5 mg/kg (*P* < 0.001), and 10 mg/kg (*P* < 0.001) Maltoamide F in homogenates of cotton ball granuloma tissues of cotton-pellet-induced rats (Figures 6(a)–6(c)).

## 4. Discussion

Inflammation is defined as a part of the host defense system and caused by varied stimuli [1, 32]. It is widely implicated in the progression of human diseases, thereby becoming the focus of scientific research [33, 34]. Recent data from anti-inflammatory assays have indicated that malyngamides are active, and Malyngamide F acetate is the most potent malyngamide with minimal cytotoxicity [22]. Here, the result of the acute toxicity test displayed that the maximum oral dose of Maltoamide F was 50 mg/kg, and it is safe at oral doses of 1–10 mg/kg for rats.

Additionally, the emerging literature has proved that malyngamides possess anti-inflammatory activity [22, 23]. For example, Villa et al. have suggested that Malyngamide F acetate shows anti-inflammatory activity in the NO assay [22]. Malloy et al. have revealed that Malyngamide 2 exerts an anti-inflammatory role in LPS-induced macrophage cells [23]. In the present study, our findings were as follows: (1) Maltoamide F notably reduced carrageenan-induced edema percentage of rat paws compared with the control group. (2) Maltoamide F suppressed increase of ear thickness compared with the control group in AA-induced rats. (3) In contrast to the control group, Maltoamide F reduced weight of ear edema in xylene-induced rats. (4) The granuloma weight was significantly diminished by Maltoamide F in cotton-pellet-induced rats. Based on these results, we inferred that Malyngamide F possessed anti-inflammatory potent in both rat models of inflammation.

To our knowledge, proinflammatory cytokines such as TNF-*α* and IL-6 exert critical roles in the inflammatory process [30]. A previous document has revealed that Malyngamide F can suppress the secretion of IL-6 and TNF-*α* in LPS-induced murine macrophages [22]. Similarly, we found that IL-6 and TNF-*α* levels were reduced by Malyngamide F (1 mg/kg, 5 mg/kg, and 10 mg/kg) in rat models of inflammation induced by carrageenan, formalin, AA, xylene, and cotton pellet. Based on these findings, we inferred that the anti-inflammation of Malyngamide F may be mediated by suppressing the production of IL-6 and TNF-*α*. On the other hand, convincing evidence has indicated that PGE-2 can cause abdominal constriction, and it is mostly responsible for causing inflammatory pain [35, 36]. Several flavonoids have been demonstrated to produce analgesic action through suppression of PGE-2 synthesis [37, 38]. In the current study, we discovered that Malyngamide F (1 mg/kg, 5 mg/kg, and 10 mg/kg) reduced the production of PGE-2 in rat models of inflammation induced by carrageenan, formalin, AA, xylene, and cotton pellet. Thus, we speculated that Malyngamide F might have analgesic activity.

## 5. Conclusions

To conclude, the present study demonstrated that Malyngamide F showed potent anti-inflammatory and antinociceptive activity in rat models of inflammation. However, more biological tests and phytochemical assays are essential to verify the anti-inflammatory and analgesic activity of Malyngamide F. Moreover, further studies will be needed to explore the precise mechanisms of Malyngamide F on antinociceptive and anti-inflammatory activities. Overall, Malyngamide F may be a potential chemotherapeutic agent in inflammation and pain treatment.

## Figures and Tables

**Table 1:** Effect of Malyngamide F on cotton-pellet-induced granuloma in rats.

Group	Granuloma weight (mg)	Inhibition of granuloma (%)
Control (1% EtOH)	38.26 ± 2.08	
Indomethacin (5 mg/kg)	12.54 ± 1.52^a^	67.22
Malyngamide F (1 mg/kg)	27.65 ± 2.13^a^	27.73
Malyngamide F (5 mg/kg)	22.46 ± 2.35^a^	52.61
Malyngamide F (10 mg/kg)	18.13 ± 1.67^a^	41.30

*Note.* a: *P* < 0.05.

## Data Availability

The datasets used and/or analyzed during the current study are available from the corresponding author on reasonable request.
